# A Rare Bacteremia in a Burn Patient: A Case Report of Achromobacter Xylosoxidans and Denitrificans

**DOI:** 10.7759/cureus.45909

**Published:** 2023-09-25

**Authors:** Saadet Özer, Merve Akin, Ahmet Cinar Yasti

**Affiliations:** 1 Department of General Surgery, Şehit Sait Ertürk Devlet Hastanesi, Ankara, TUR; 2 Department of General Surgery, Ankara Bilkent City Hospital, Ankara, TUR; 3 Department of General Surgery, Health Sciences University, Medical School, Ankara, TUR

**Keywords:** gram-negative sepsis, greft lysis, bacteraemia, burns, achronomobacter

## Abstract

*Achromobacter* is a rare bacteria that causes bacteremia in immune-compromised patients. In this case, a 26-year-old male patient with major burns with a positive blood culture for *Achromobacter* is presented. A rare bacterium, *Achromobacter* *xylosoxidans*, and *denitrificans*, was detected in the blood culture of a patient who was hospitalized due to major burn trauma and had delayed wound healing and had no graft take. After treatment with culture-specific antibiotics, the patients' acute phase reactants decreased, and he was discharged with 100% graft-take. Sepsis is the most common cause of death in major burns. Endogenous and exogenous bacteria cause sepsis. Bacteremia and sepsis are the most important factors affecting wound healing in burn patients. This case shows that rare opportunistic bacteria such as *Achromobacter spp.* should be considered in major burn patients with delayed wound healing and recurrent graft lysis.

## Introduction

In burn patients admitted to the hospital, infection is a significant cause of morbidity and mortality [1]. Gram-negative bacteria, predominantly in burn surgery, can lead to life-threatening infections in hospitalized immune-compromised patients [2].

Burn wounds rapidly become colonized with microorganisms, including gram-negative bacteria, gram-positive bacteria, and/or yeasts, such as those from the patient's endogenous gastrointestinal and upper respiratory tract flora, hospital flora, or the hands of healthcare workers, within the first few days [3].

In burn patients, the following microorganisms with multiple antibiotic resistance are frequently implicated as causative agents of infection: methicillin-resistant *Staphylococcus aureus* (MRSA), vancomycin-resistant *enterococci*, *Pseudomonas aeruginosa*, *Klebsiella pneumoniae*, *Escherichia coli*, *Acinetobacter spp.*, various members of the *Enterobacteriaceae* family, and *Bacteroides* species [3].

*Achromobacter xylosoxidans* was first isolated and described by Yabuuchi and Ohyama in 1971. It belongs to the *Alcaligenaceae* family and is an aerobic, motile, oxidase-positive, non-fermenting, gram-negative bacillus [4,5]. In 1998, it was classified into two different subspecies, *Achromobacter xylosoxidans* and *Achromobacter denitrificans* [5]. It is an opportunistic bacterium that thrives in aqueous and moist environments, such as respiratory equipment, incubators, and disinfectant solutions [2,6]. It has been found to cause infections, particularly in immune-compromised and neutropenic patients in hospitals [2]. The microorganism can lead to clinical infections such as pneumonia, bacteremia, and meningitis [4]. Its treatment is challenging due to intrinsic and acquired resistance mechanisms [5,7].

Currently, early excision and graft application are presented as the gold standard in the treatment of burn patients. Early intervention is important, especially in major burns, as the lack of a skin barrier causes rapid and progressive infection and even mortality [8]. The successful graft removal rate is directly proportional to the absence of local and systemic infections. In unsuccessful grafting operations, the first thing that comes to mind is wound infection.

In this case report, a patient whose wound healing was delayed and repeated grafting failed and whose blood culture was *Achromobacter* positive is presented.

## Case presentation

A 26-year-old male patient presented to a burn treatment center due to a flame burn injury. At the time of admission, the patient had a 35% burned total body surface area (TBSA), affecting both the lower and upper extremities, hands, and face. He underwent repeated escharectomy and graft procedures at the burn center, but due to unsuccessful grafting, he was referred to our clinic.

On the 26th day after the burn injury, the patient was transferred to our center. Upon admission to our center, the patient had non-epithelial burn wounds covering 25% of the total body surface area. It was observed that 10% of the total body surface area was grafted, but there was almost complete graft lysis in the grafted areas.

In our clinic, patients are accepted from external centers according to clinical protocols, blood and urine cultures are performed, and wound dressings are changed within the first hour. For major burn patients being monitored and treated in the intensive care unit of our center, in order to detect bacteremia before the septicemia stage, blood and urine cultures are repeated twice a week.

Immediately after admission to our center, the patient's blood and urine cultures were taken, and complete blood count (CBC), C-reactive protein (CRP), lactate values, renal and hepatic functions were studied. Intravenous Ceftriaxone 2x1g and Metronidazole 3x500mg were started empirically in consultation with the Infectious Diseases Clinic. On the third day of admission to our center, *Achromobacter denitrificans* were reported in the blood cultures taken upon admission. Although acute-phase reactants decreased with empirical antibiotics, the antibiotic therapy was revised, and Imipenem 4x500mg was initiated. Repeated blood cultures are still positive for *Achromobacter denitrificans* and *Achromobacter xylosoxidans*. Intravenous Piperacilin Tazobactam 4x4.5 g treatment was started for the patient, whose CRP level continued to be elevated despite the negative blood culture under imipenem treatment, upon the recommendation of the Infectious Diseases Clinic. The blood culture examination was continued every two days, and there was no decrease in CRP or other acute phase reactants in the patient. On the 20th day of hospitalization, the blood culture was positive for *Staphylococcus haemolyticus* and *Acinetobacter baumannii*, and intravenous Tigecycline 2x50 mg and Polymyxin B 2x1 million IU were started. This treatment protocol was continued for 15 days upon the recommendation of the Infectious Diseases Clinic. Table 1 summarizes the laboratory findings, blood cultures, and treatment. After three consecutive blood and urine cultures turned out negative, the acute phase reactant decreased, and antibiotic therapy was discontinued. The patient's infection was monitored by tracking WBC, neutrophil/lymphocyte ratio, platelet, Delta neutrophile index (DNI), albumin, lactate dehydrogenase (LDH), lactate, and C-reactive protein (CRP) values.

**Table 1:** Timeline of blood samples, culture results and antibiotics WBC: White blood cell, N/L: Neutrophile lymphocyte ratio, PLT: Platelet, DNI: Delta neutrophile index, ALB: Albumin, LDH: Lactate dehydrogenase, LAC: Lactate, CRP: C-reactive protein, IU: International unit

Date	WBC 10^9/L	N/L	PLT 10^9/L	DNI %	ALB g/L	LDH U/L	LAC mmol/L	CRP mg/L	Blood Culture	Urine Culture	Antibiotic
10.2	26.61	16,92	632	3.8	30	278	4.4	79	Achromobacter denitrificans	Culture (-)	Seftriakson 2x1 gr Metranidasol 3x500 mg
11.2	15.95	6,83	533	<0.1	27	227	1.5	82			
13.2	11.86	15	715	1.3	25	206	0.8	30	Achromobacter denitrificans	Culture(-)	Seftriakson 2x1 gr Metranidasol 3x500 mg
15.2	5.22	7.25	432	4.1	24	314	0.4	25	Achromobacter xylosoxidans	Culture (-)	Imıpenem 4x500
19.2	8.64	3.44	816	<0.1	23	238	0.9	68			
20.2	5.70	3.88	485	5.5	20	183	1.6	65	Culture (-)	Candida albicans	Imıpenem 4x500
21.2	3.80	1.47	555	4.3	20	192	2.3	94			
23.2	4.93	1.59	376	2.1	21	173	2.4	83	Culture (-)	Candida albicans	Piperacilin Tazobactam 4x4.5 gr
24.2	4.65	3.02	324	5.7	21	157	3.0	92			
25.2	4.04	1.73	275	<0.1	22	122	1.3	118			
27.2	4.34	1.94	254	<0.1	21	130	1.2	50	Culture (-)	Culture (-)	Piperacilin Tazobactam 4x4.5 gr
2.3	2.34	0.93	266	<0.1	22	98	3.3	73	Staphylococcus haemolyticus	Culture (-)	Piperacilin Tazobactam 4x4.5 gr
3.3	3.46	1.46	323	7.2	22	155	2.4	85			
7.3	4.24	4.2	285	<0.1	24	112	5.9	56	Acinetobacter baumannii	Culture (-)	Piperacilin Tazobactam 4x4.5 gr
9.3	3.71	1.92	296	<0.1	23	102	1.6	67	Culture (-)	Candida parapisilozis	Piperacilin Tazobactam 4x4.5 gr
11.3	5.08	1.46	315	<0.1	25	159	1.3	57	Culture (-)	Culture (-)	Tigesiklin 2x50 mg Polimiksin B 2x1 milion IU
17.3	5.00	1.55	332	<0.1	23	135	1.4	61	Culture (-)	Culture (-)	Tigesiklin 2x50 mg Polimiksin B 2x1 milion IU
20.3	4.73	2.55	316	<0.1	23	139	1.2	24	Culture (-)	Culture (-)	Tigesiklin 2x50 mg Polimiksin B 2x1 milion IU

On the 26th day after the burn injury, when the patient was admitted, non-epithelialized burn wounds covered a total of 25% of the TBSA. Due to the high WBC and CRP values, graft lysis, and moderate exudation in the wounds, infection was suspected in the burn wounds, and silver dressings were continued for wound care. Subsequently, as the blood cultures were positive for *Achromobacter*, previously planned grafting of the burn areas was postponed until the 22nd day of admission. This decision was made due to graft lysis and the continuation of clinical signs of infection. After using appropriate antibiotics based on culture and sensitivity and the negative result of the blood culture, it was considered that the wound site was suitable for grafting, and grafting was performed in seven sessions starting from the 22nd day of admission to our center. Through consecutive operations, all burn areas were grafted, and graft takes was 100% successful.

After admission, the patient received continuous physiotherapy to maintain joint range of motion. The patient was provided with a routine high-protein diet at the hospital, and in addition to the meals, he was supplemented with 20mg/day of glutamine and a high-protein (1.5g/ml) enteral nutrition solution. On the 85th day after the burn injury and the 59th day of admission to our clinic, the patient was discharged in a healed condition.

## Discussion

In cases of major burns, inflammatory markers such as CRP, WBC, and ferritin usually elevate in almost every patient during the natural course of burn shock [9]. Clinical studies have demonstrated a relationship between these inflammatory markers and mortality [10]. However, these markers also serve as indicators of sepsis, which complicates the diagnosis of sepsis in burn patients [11,12].

Mortality in major burns occurs in two stages. If death does not occur due to burn shock, deaths related to sepsis may develop within weeks. Early diagnosis and treatment are the most effective methods for preventing sepsis-related deaths. In cases where sepsis markers are already positive, early detection of bacteremia and the initiation of appropriate antibiotic treatment are crucial [13].

Sepsis in major burn patients most commonly develops due to the absence of the skin barrier, leading to bacteremia caused by normal skin and intestinal flora bacteria. Therefore, in patients with major burns, routine blood and urine cultures are taken every 72 hours for monitoring, as it is considered a preventive measure in our center.

In this presented case, upon admission to our clinic, the inflammatory markers (WBC, CRP, DNI, and Lactate) were elevated. The patient was admitted on the 26th day after the burn trauma, and their vital signs were stable. Although sepsis was not initially suspected, the lack of graft take; and the presence of elevated inflammatory markers raised concerns about possible bacteremia in the patient (Table 1). Subsequently, a blood culture was obtained, which resulted in the isolation of *Achromobacter xylosoxidans* and *denitrificans*.

In a retrospective study conducted by Schulz et al., they monitored 685 patients in the intensive care unit over a period of 10 years. Among these patients, *Achromobacter xylosoxidans* was isolated in cultures from 20 patients. The bacteria were found in wound cultures of 16 patients, blood cultures of one patient, urethral cultures of one patient, and tracheal cultures of two patients [2].

*Achromobacter xylosoxidans* is a rare and life-threatening microorganism that causes serious bacteremia in immune-compromised patients. It can lead to a clinical presentation that is indistinguishable from other gram-negative bacterial sepsis. The most common clinical conditions associated with *Achromobacter xylosoxidans* infections are bacteremia and pneumonia. Less frequently, it can cause skin and soft tissue infections and surgical site infections [7]. Although burned patients are accepted as immune-compromised, this is the first *Achromobacter* bacteremia seen at our 20-year-old center.

In studies conducted, *Achromobacter xylosoxidans* has been found to exhibit resistance to many antibiotics, including carbapenems, amoxicillin, first, and second generation cephalosporins, and aminoglycosides, due to various intrinsic and acquired resistance mechanisms. However, it has been determined that it remains susceptible to certain antibiotics, such as piperacillin, tigecycline, colistin, and doxycycline [14].

In the case presented, the patient was referred to our center from an external facility due to unsuccessful repeated grafting procedures. Upon admission, a blood culture was taken, which revealed the presence of *Achromobacter*. After initiating antibiotic treatment based on the sensitivity of *Achromobacter* to specific antibiotics (Table 1), the blood culture turned negative. Following this eradication of *Achromobacter*, grafting was performed, and 100% graft take was achieved. In the literature, a positive blood culture for *Achromobacter* in a burn patient has been reported in only one case [2]. However, the impact of bacteremia on graft take was not mentioned in that case. According to the best of our knowledge, our presented case is the first study to associate *Achromobacter bacteremia* with graft-take success. In our presented case, it was considered that the unsuccessful grafting in the external facility might have been due to *Achromobacter bacteremia*. After eradication of *Achromobacter* and other microorganisms was achieved, the patient's treatment was completed with 100% graft-take.

## Conclusions

*Achromobacter xylosoxidans* and *denitrificans* is a rare bacterium isolated from blood cultures of burn patients monitored in intensive care units. The clinical presentation is similar to sepsis caused by other gram-negative bacteria. In major burns, if there are difficulties in wound management during the acute phase, wound infection is usually the first consideration. However, as seen in this case, infections caused by rare pathogens can also decrease wound healing and graft take success. Therefore, in patients who undergo repeated graft procedures with unsuccessful graft take, the possibility of colonization by rare and resistant gram-negative bacteria like *Achromobacter* species should be considered, and coordination with microbiology laboratories should be established accordingly.
